# Do Parental Psychopathology and Higher‐Order Beliefs Predict Symptom Changes in Young People Following Metacognitive Therapy?

**DOI:** 10.1002/cpp.70122

**Published:** 2025-07-19

**Authors:** Anne Thingbak, Jennifer L. Hudson, Adrian Wells, Mia Skytte O'Toole

**Affiliations:** ^1^ Department of Psychology and Behavioural Sciences Aarhus University Aarhus Denmark; ^2^ Black Dog Institute University of New South Wales Sydney NSW Australia; ^3^ University of Manchester School of Psychological Sciences & Greater Manchester Mental Health NHS Trust Manchester UK

**Keywords:** adolescents, anxiety, children, depression, metacognitive therapy, parental beliefs

## Abstract

Preliminary evidence indicates that metacognitive therapy (MCT) is a promising treatment in children and adolescents; however, little is known about the influence of parental factors on MCT outcomes. Based on secondary analyses from a recent trial of MCT in young people with anxiety and depressive disorders, the aim of this study was to evaluate cross‐sectional associations between child and parental symptoms, metacognitive beliefs and attention control beliefs and whether such parent variables predicted symptom scores following MCT. The sample consisted of 97 children and adolescents aged 10–17 years (*M* = 12.85 ± 1.9, 82.5% females) and their parents (*n* = 145, 57.9% mothers). The majority of the sample had a primary anxiety disorder (*n* = 96). Participants received eight sessions of MCT in transdiagnostic groups. The majority of baseline correlations were small and non‐significant with some exceptions. Small significant relationships emerged between maternal attention shifting and child attention shifting, total attention control and symptoms. Also, child depressive symptoms were associated with maternal anxiety, maternal cognitive self‐consciousness, and paternal attention control beliefs, with small to moderate effects. Parental factors did not predict symptom scores at post‐treatment nor at 6‐month follow‐up. The findings should be considered preliminary and seen in light of the overall good mental health of the parents. If results are replicated in the future with broader samples, it suggests that MCT outcomes in young people may have little association with the symptoms and higher‐order beliefs of parents.

**Trial Registration:** AsPredicted number: 152970

## Summary

- Little is known about the effects of parental factors on MCT outcomes.
- We evaluated relationships between child and parental psychopathology, metacognitive beliefs, and attention control beliefs.
- Results revealed predominantly small and non‐significant baseline associations between child and parent measures.
- Significant baseline associations were found between specific child and maternal symptoms and attention control beliefs; and between child symptoms and specific higher‐order beliefs of parents.
- Parental factors did not predict changes in symptoms or diagnostic status following MCT.

## 1 Introduction

Anxiety and depressive disorders affect 6.5% and 2.6%, respectively, of children and adolescents worldwide (Polanczyk et al. 2015) and often co‐occur (Melton et al. 2016). If left untreated, they are associated with increased difficulties on a range of outcomes in life (including mental and physical health, education and social functioning) and with substantial family and societal economic costs (Copeland et al. 2021; de Lijster et al. 2018; Pollard et al. 2023). Cognitive behavioural therapy (CBT) is recommended as a first‐line treatment (e.g., National Institute for Health and Care Excellence [NICE] 2013, 2019), but only approximately 50% of children with anxiety remit from their primary disorder following CBT (James et al. 2020). Similarly, the response rate is approximately 40% in children with a clinical or subclinical depressive disorder (Cuijpers et al. 2023). In adults, metacognitive therapy (MCT; Wells 2009) has been established as an effective, evidence‐based treatment, with meta‐analyses demonstrating large between‐group differences favouring MCT when compared with CBT at post‐treatment (e.g., Andersson et al. 2025). Building on adult research, preliminary evidence has emerged indicating that MCT is also a promising treatment for young people (Akhouri and Madiha 2022; Esbjørn et al. 2018; Reinholdt‐Dunne et al. 2024; Thingbak et al. 2024b; Thorslund et al. 2020).

MCT has its origins in a scientifically derived model of emotional disorder, the Self‐Regulatory Executive Function (S‐REF) model (Wells and Matthews 1994, 1996). The S‐REF model proposes that anxiety and depression derive from biases in metacognition (i.e., the part of cognition that is involved in the monitoring, appraisal and control of first‐order cognition) and in volitional attention control (i.e., the ability to exert voluntary control over attention). According to the model, these biases influence top‐down selection and implementation of self‐regulation strategies that prolong rather than terminate negative processing and maintain psychological distress (Wells and Matthews 1994, 1996). For example, metacognitive beliefs that worry is helpful for avoiding danger and/or beliefs that thinking/attention is uncontrollable are incompatible with disengaging from repetitive negative processing. In the model, attention control comprises *actual* attentional performance skills, which are differentiated from *beliefs* about attention, supported by evidence that behavioural and self‐reported attention control are only weakly associated and appear to relate differently to measures of psychopathology (Quigley et al. 2017; Van Bockstaele et al. 2023). In the S‐REF model, attention control beliefs (e.g., the perceived ability to disengage from perseverative thinking and shift one's focus of attention) are a part of a broader metacognitive knowledge system facilitating cognitive regulation (Wells 2019). There is substantial evidence supporting central tenets of the S‐REF model in young populations (e.g., Belte et al. 2024; Reinholdt‐Dunne et al. 2019; Schultz et al. 2023; Thingbak et al. 2024a), but less is known about the effects of MCT in youth and the factors that correlate with treatment outcomes.

Previous research on the transgenerational transmission of psychopathology indicates that children of parents with an anxiety disorder are significantly more likely to develop anxiety disorders (risk ratio: 1.76) and depressive disorders (risk ratio: 1.31) than children of parents without anxiety disorders (Lawrence et al. 2019). Moreover, elevated parental psychopathology has, along with other factors (e.g., higher symptom severity, comorbidity and a diagnosis of social anxiety), been identified as a robust predictor of poorer treatment outcomes in CBT for child anxiety (Bertie et al. 2024a, 2024b; Hudson et al. 2015; Kunas et al. 2021). Several factors have been proposed as an explanation for the link between child and parental psychopathology, with psychological theories highlighting the potential role of parental behaviours (e.g., information transfer, modelling, rejection and overcontrol) that may increase the child's perception of threat and limit opportunities to build confidence in their ability to cope (Rapee et al. 2023). Meta‐analyses have linked internalizing problems in children with inter‐parental conflict, aversiveness, lack of warmth, abuse and over‐involvement (Yap and Jorm 2015); parental control (Chyung et al. 2022; Van Der Bruggen et al. 2008); and parental cognitions and expectations about child anxiety (Fox and Fleming 2025).

It is possible that the association between suboptimal parental behaviour and internalizing problems in children is mediated by cognitive factors (e.g., Gallagher and Cartwright‐Hatton 2008; McGinn et al. 2005). For example, a study by Gallagher and Cartwright‐Hatton (2008) indicates that overreactive parenting (i.e., harsh, punitive and inconsistent discipline styles) contribute to the development of cognitive distortions and biased metacognitive beliefs and that the child's cognitive style partially mediates the association between overreactive parenting and child anxiety. Additionally, parental criticism, rejection and discouragement have been associated with biased metacognitions about the usefulness of rumination in adolescents (Chow and Lo 2017). Studies have also demonstrated a more direct link between the dysfunctional cognitive style held by children and parents, for instance, in terms of interpretation bias (Subar and Rozenman 2021) and intolerance of uncertainty (Yao and Kishimoto 2025). As such, it is possible that children's metacognitive beliefs or knowledge about cognition and attention as implicated in the S‐REF model are associated with the beliefs held by their parents. Specifically, just like parents provide information about the environment to their children, it is possible that parents also provide information about internal events, that is, how to interpret and respond to thoughts and feelings. It has previously been suggested that whereas parents may be unlikely to express their metacognitions explicitly (e.g., ‘You need to worry in order to be prepared’), parents may express such beliefs indirectly through verbal communication and discussions around worries and rumination (Esbjørn et al. 2016; Wilson et al. 2011). For instance, anticipating potential outcomes of a feared situation might reinforce metacognitive beliefs such as ‘worrying will help me cope’, whilst valuing analyzing situations in the past could have a similar effect supporting beliefs such as ‘analyzing will give me answers’. Furthermore, information about unpleasant thoughts (e.g., ‘it is bad to think certain thoughts’) may be potential pathways to internalizing higher‐order cognitions.

Several studies have investigated potential associations between child and parental metacognitive beliefs. In community samples of children, two studies have found non‐significant associations of negligible or small effects (*r*s = −0.12–0.27, age: 8–16; Donovan et al. 2017; Wilson et al. 2011), three studies have found significant associations of small to medium effects (*r*s = 0.14–0.38, age: 7–16; Chow and Lo 2017; Esbjørn et al. 2016; Lønfeldt et al. 2017), and one study has found significant associations of medium to large effects (*r*s = 0.38–0.54, age: 8–16; Köcher et al. 2023). In clinical samples of children, preliminary results also suggest associations of small effects (*r*s = 0.18–0.24, age: 8–16; Köcher et al. 2023). However, no significant differences have been found in the levels of metacognitive beliefs held by parents of children with and without anxiety disorders (Donovan et al. 2016). Only one study has investigated the relationships between child and parental metacognitive beliefs over time, finding that maternal metacognitions were associated with child metacognitions and anxiety levels over a 3‐year period of small effects (*r*s = 0.20–0.25; Walczak et al. 2021). Moreover, maternal baseline metacognitions significantly predicted child metacognitions at follow‐up but did not predict child anxiety when children's metacognitions were taken into account (Walczak et al. 2021). In sum, there is some preliminary evidence suggesting reliable but modest positive associations between children's and parents' metacognitive beliefs. However, research to date is limited by primarily focusing on non‐clinical samples of children and an overrepresentation of mothers in parent samples (range: 74%–100%). Thus, studies investigating clinical samples and including both mothers and fathers are warranted. Moreover, no study to date has investigated whether parental psychopathology and parental higher‐order beliefs are associated with symptom changes in young people following MCT. An evaluation of parental correlates would be an important step in understanding potential influences on symptoms following treatment. The present study was based on data from a recent uncontrolled trial of group MCT for children and adolescents aged 10–17 years (Thingbak et al. 2024b). It had two aims: (1) to investigate associations between child and parental symptoms of anxiety and depression, metacognitive beliefs and attention control beliefs in a clinical sample of children and their parents and (2) to investigate whether parental symptoms, metacognitive beliefs and attention control beliefs (measured at pre‐treatment) predicted symptom changes immediately following treatment and through a 6‐month follow‐up period. Given the scarcity of research on fathers, we explored the role of mothers and fathers separately. Furthermore, as the transition from childhood to adolescence marks a potential shift in the role of parents (e.g., Lønfeldt et al. 2017; Rapee et al. 2023), it is possible that the role of parental factors differs depending on the age of the child. Therefore, we aimed to explore age as a moderator of the above‐mentioned relationships, that is, whether baseline associations as well as the predictive effect of parent measures varied depending on the age of the child.

## 2 Methods

The first part of this study (i.e., cross‐sectional child–parent associations) was preregistered with the regional ethical committee (#1‐10‐72‐217‐21) and was later made publicly available at AsPredicted (#152970) with no changes made. The second part (i.e., predictive effect of parent measures) was preregistered at AsPredicted (#152970)^1^.

### 2.1 Participants and Procedure

Participants in the current study took part in a larger project investigating group MCT for children and adolescents (Thingbak et al. 2024b). Families were self‐referred and received the treatment free of charge. The child sample consisted of 97 young people aged 10–17 years (*M* = 12.85, *SD* = 1.9, 82.5% females) presenting with a primary DSM‐5 anxiety or depressive disorder (American Psychiatric Association 2013). The majority of children presented with a primary anxiety disorder (99.0%, *n* = 96), whereas one child (1.0%) presented with a primary depressive disorder. An additional 9.3% (*n* = 9) of children presented with a comorbid depressive disorder. For the purpose of the present study, a minimum of one parent was invited to participate along with each child by completing online self‐report measures at baseline. A total of 145 parents (84 mothers, 61 fathers) agreed to participate. Non‐participating parents were still encouraged to take part in the treatment programme. Children were diagnostically assessed at baseline and again 2 weeks following treatment. At baseline, both children and parents reported on their symptoms of anxiety and depression, metacognitive beliefs and attention control beliefs. Moreover, children reported on their symptoms, metacognitive beliefs and attention control beliefs weekly throughout treatment and again at post‐treatment and at 3‐ and 6‐month follow‐up. All parents and children ≥ 15 years of age gave written informed consent, and children < 15 years of age orally assented to participate.

The treatment consisted of eight weekly sessions of MCT in transdiagnostic groups. It followed the adult MCT manual for generalized anxiety by Wells (2009) with slight adjustments to ensure age‐appropriate language and metaphors. Aligning with Esbjørn et al. (2018), attention training (Wells 1990, 2019) was included to provide children with a concrete way of understanding voluntary attention control and thus, their ability to disengage from perseverative thinking and self‐focused attention. The final 30 min of each session was allocated to the parents. They were socialized to the treatment rationale and instructed on how to support their child. For instance, parents were instructed to help their child postpone worry and rumination and reallocate their attention. Also, they were instructed to encourage children to reduce avoidance strategies.

### 2.2 Child Measures

*Anxiety Disorders Interview Schedule: Child and Parent Versions* (ADIS‐IV‐C/P; Silverman and Albano 1996) is a diagnostic interview conducted with children and their parents, separately. It assesses the presence of child anxiety, depression and related disorders according to the DSM‐IV. A diagnostic status and clinical severity were evaluated by the assessor based on a composite rating of both child and parent interviews. To align with the most recent version of the DSM (i.e., DSM‐5; American Psychiatric Association 2013), obsessive compulsive disorder (OCD) was not classified as an anxiety disorder. Diagnostic interviews at intake were conducted in‐person by the first author, who is a clinical psychologist. At post‐treatment, interviews were conducted online via video call by master's students in psychology, who were trained in conducting diagnostic interviews and receiving supervision from the first author. All interviews were video recorded in order to assess inter‐rater agreement on clinical diagnoses. Inter‐rater agreement on assigned diagnoses before and after treatment was excellent (kappa: pre‐treatment = 0.93, post‐treatment = 1.00).

*Revised Anxiety and Depression Scale—Child Version* (RCADS‐C; Chorpita et al. 2000) is a 47‐item self‐report measure assessing symptoms of anxiety and depression according to the DSM‐IV criteria. The scale consists of six subscales: generalized anxiety disorder, separation anxiety disorder, panic disorder, social phobia, OCD and major depressive disorder. Items are rated on a 4‐point scale from 0 (*never*) to 3 (*always*). Higher scores indicate greater symptom severity. In the present sample, Cronbach's α was 0.93 (subscales = 0.72–0.91).

*The Metacognitions Questionnaire—Adolescent Version* (MCQ‐A; Cartwright‐Hatton et al. 2004) is a 30‐item self‐report measure (slightly adapted from the MCQ‐30; Wells and Cartwright‐Hatton 2004) assessing metacognitive beliefs in youth across five subscales: positive beliefs about worry (POS), negative beliefs about worry (NEG), cognitive confidence (CC), need to control thoughts (NC) and cognitive self‐consciousness (CSC). Items are rated on a 4‐point scale ranging from 1 (*do not agree*) to 4 (*agree very much*). Higher scores indicate greater levels of maladaptive metacognitions. In this study, Cronbach's α was 0.92 (subscales = 0.79–0.87).

*The Attentional Control Scale for Children* (ACS‐C; Derryberry and Reed 2002; Muris et al. 2004) is a 20‐item self‐report measure assessing perceived attentional control in children. The ACS‐C consists of two subscales: attention focusing and attention shifting. Items are rated on a 4‐point scale ranging from 1 (*almost never*) to 4 (*always*). After recoding reversed items, higher total scores indicate better attention control. Cronbach's α in the present study was 0.85 (subscales = 0.77).

### 2.3 Parent Measures

*The Hospital Anxiety and Depression Scale* (HADS; Zigmond and Snaith 1983) is a 14‐item self‐report measure assessing symptoms of anxiety and depression. Across two subscales (anxiety and depression), items are rated on a 4‐point scale ranging from 0 to 3. Higher scores indicate greater levels of symptoms. Cronbach's α was good (mothers = 0.85, fathers = 0.88).

*The Metacognitions Questionnaire—Short Form* (MCQ‐30; Wells and Cartwright‐Hatton 2004) is a 30‐item self‐report measure assessing metacognitive beliefs across five subscales: positive beliefs about worry (POS), negative beliefs about worry (NEG), cognitive confidence (CC), need to control thoughts (NC) and cognitive self‐consciousness (CSC). Items are rated on a 4‐point scale ranging from 1 (*do not agree*) to 4 (*agree very much*). Higher scores indicate greater levels of maladaptive metacognitions. Cronbach's α was good (mothers = 0.93, fathers = 0.88).

*The Attention Control Scale* (ACS; Derryberry and Reed 2002) is a 20‐item self‐report measure assessing attentional control across two subscales: attention focusing and attention shifting. Items are rated on a 4‐point scale ranging from 1 (*almost never*) to 4 (*always*). After recoding reversed items, higher total scores indicate better perceived attention control. Cronbach's α was good (mothers = 0.91, fathers = 0.85).

### 2.4 Analytic Strategy

First, bivariate correlations were computed to explore cross‐sectional relationships between child and parental symptoms, metacognitive beliefs and attention control beliefs at baseline. Parent variables were investigated separately for mothers and fathers. Effect sizes were expressed as Pearson's *r* with 0.1, 0.3 and 0.5 considered small, medium and large effects, respectively (Cohen 1988). Second, multilevel models (MLMs) were chosen to determine whether parental symptoms, metacognitive beliefs and attention control beliefs served as predictors of symptoms at post‐treatment and 6‐month follow‐up, respectively. The primary outcome measure at both time points was children's self‐reported symptoms of anxiety and depression (RCADS total score), whereas the secondary outcome measure was remission from primary diagnosis based on ADIS‐IV‐C/P (post‐treatment only). MLMs were chosen as these models tolerate missing observation points (Chakraborty and Gu 2019). Missing data at the item level was not an issue as the measures of interest were filled out online with forced answer. In the case of premature termination of a questionnaire with less than 50% items completed, participants were excluded. If more than 50% items were completed, missing items were handled with mean substitution (Schafer and Graham 2002). Data were hierarchically arranged in two levels, with time as a continuous variable at Level 1 nested within individuals at Level 2. The proposed predictors of treatment effects, that is, parental symptoms of anxiety and depression (HADS), metacognitive beliefs (MCQ‐30) and attention control beliefs (ACS), were investigated in separate models. For the primary outcome, analyses were conducted based on the intention‐to‐treat principle (i.e., all participants). Analyses from pre‐ to post‐treatment were based on nine time points (baseline, weekly throughout treatment and post‐treatment) using a linear function of time. From pre‐treatment to 6‐month follow‐up, analyses were based on the main time points (baseline, post‐treatment, and follow‐up at 3 and 6 months) using a log‐linear function of time (Thingbak et al. 2024b). Effect sizes were expressed as Cohen's *d* with values of 0.2, 0.5 and 0.8 considered small, medium and large effects (Cohen 1988). For the secondary outcome, logistic regression analyses were conducted investigating the predictive role of baseline parent variables on children's diagnostic status at post‐treatment (remitted vs. not remitted). Effect sizes were expressed as Pearson's *r* with 0.1, 0.3, and 0.5 considered small, medium, and large effects (Cohen 1988). Finally, the effect of age on the above relationships, that is, baseline associations and the predictive effects of parent measures, was explored in interaction analyses (time*parent variable*age).

## 3 Results

### 3.1 Descriptive Statistics

All participating children and parents filled out all questionnaires at baseline, with the only exception of two missing items for fathers. Baseline demographics and clinical characteristics are presented in Table 1. The majority of parent units were cohabiting (74.2% of all units, *n* = 72) and nearly all parents were born in Denmark (91.1%; *n* = 175). The majority of both mothers (85.7%, *n* = 72) and fathers (75.4%, *n* = 46) who filled out baseline measures had a medium or long academic education. Within a year prior to participating in the study, 29.8% (*n* = 25) of mothers and 18.0% (*n* = 11) of fathers reported having experienced psychological problems. At baseline, parents' self‐reported symptoms of anxiety and depression (mothers: *M* = 2.8–5.6; fathers, *M* = 2.9–4.7) fell within the normal range of 0–7 (Zigmond and Snaith 1983). Significant group differences were observed between mothers and fathers on the MCQ total score, negative beliefs about worrying (NEG), need to control thoughts (NC) and attention focusing beliefs (*p*s < 0.05). Table 2 displays means and standard deviations for parent and child measures.

**TABLE 1:** Baseline demographics and child clinical characteristics.

Children	
Age range (*M* ± *SD*)	10–17 (12.85 ± 1.9)
% female	82.5% (*n* = 80)
Country of birth	Denmark (97%, *n* = 94) Sweden (2%, *n* = 2) Belgium (1%, *n* = 1)
Primary disorders	Separation anxiety (7.2%, *n* = 7) Social anxiety (26.8%, *n* = 26) Specific phobia (2.1%, *n* = 2) Panic disorder (1%, *n* = 1) Panic disorder with agoraphobia (3.1%, *n* = 3) Agoraphobia (5.2%, *n* = 5) Generalized anxiety disorder (53.6%, *n* = 52) Persistent depressive disorder (dysthymia; 1%, *n* = 1)
CSR of primary disorder, *M* (*SD*)	5.4 (*SD* ± 0.76)
Mean number of anxiety disorders	1.82 (*SD* ± 0.83, range 0–3)
Mean number of anxiety and depressive disorders	1.91 (*SD* ± 0.82, range 1–4)
Comorbid disorders	Separation anxiety disorder (14.4%, *n* = 14) Social anxiety (22.7%, *n* = 22) Specific phobia (8.2%, *n* = 8) Panic disorder (2.1%, *n* = 2) Panic disorder with agoraphobia (3.1%, *n* = 3) Agoraphobia (7.2%, *n* = 7) Generalized anxiety disorder (23.7%, *n* = 23) Persistent depressive disorder (dysthymia; 3.1%, *n* = 3) Major depressive disorder (6.2%, *n* = 6) OCD (7.2%, *n* = 7) PTSD (1%, *n* = 1)
Psychotropic medication	1% (*n* = 1)
Previous depressive episode(s)	7.2% (*n* = 7)

Parent unit	
Country of birth, all parents (*n* = 192)	Denmark (91.1%, *n* = 175) Other European countries (4.2%, *n* = 8) Middle East (*n* = 1) Asia (*n* = 6) Africa (*n* = 1) South America (*n* = 1)
Status of parent unit (*n* = 97)	Cohabiting = 74.2% (*n* = 72) Separated = 23.7% (*n* = 23) Single parents = 2.1% (*n* = 2)
Previously reached out for psychological or psychiatric treatment for child (*n* = 97)	61.9% (*n* = 60)

Participating parents	
Education: medium or long academic, incl. PhD	Mothers: 85.7% (*n* = 72) Fathers: 75.4% (*n* = 46)
Household monthly income ≥ 65,000 DKK (≈$9500)	Mothers: 64.3% (*n* = 54) Fathers: 70.5% (*n* = 43)
Have experienced psychological problems within past year	Mothers: 29.8% (*n* = 25) Fathers: 18.0% (*n* = 11)

**TABLE 2:** Means and standard deviations for child and parent measures.

	Parents (baseline)				Children							
	Mothers (*n* = 84)		Fathers (*n* = 61)		Baseline (*n* = 97)		Post‐MCT (*n* = 87)		3‐month FU (*n* = 83)		6‐month FU (*n* = 75)	
	*M*	*SD*	*M*	*SD*	*M*	*SD*	*M*	*SD*	*M*	*SD*	*M*	*SD*
Total anx/dep^a^	8.42	5.23	7.61	5.50	57.36	21.81	30.45	20.00	34.14	24.36	30.96	22.78
Anxiety^b^	5.64	3.38	4.70	3.18	45.61	17.26	23.57	15.13	26.47	18.23	23.85	17.58
Depression^c^	2.77	2.49	2.90	2.90	11.75	5.64	6.87	6.19	7.67	6.86	7.11	6.34
MCQ total	50.87*	14.09	46.10*	10.61	62.72	15.34	40.21	8.22	42.84	11.24	41.65	9.50
MCQ POS	9.08	3.16	8.36	3.12	9.37	3.75	6.95	1.90	7.00	1.98	7.45	2.72
MCQ NEG	10.95*	4.10	9.51*	3.57	16.90	4.05	8.76	2.95	9.98	4.07	9.52	3.50
MCQ CC	10.01	3.96	9.16	3.28	10.36	4.39	8.26	3.78	8.69	4.02	8.52	4.03
MCQ NC	8.67*	2.90	7.62*	2.34	12.42	4.24	7.63	2.23	8.20	2.60	7.85	1.98
MCQ CSC	12.15	4.09	11.44	3.60	13.67	4.34	8.60	3.10	8.98	4.01	8.31	3.06
ACS total	58.11	10.07	60.77	7.58	46.80	9.81	50.60	10.17	50.78	9.98	51.52	9.91
ACS focusing	25.33*	4.90	26.90*	3.56	20.71	5.10	23.15	4.89	23.28	4.71	23.75	4.85
ACS shifting	32.77	6.11	33.87	5.29	26.09	5.76	27.45	6.17	27.50	5.98	27.77	6.09

Abbreviations: ACS = Attention Control Scale, ACS‐C = Attention Control Scale (Child Version), CC = cognitive confidence, CSC = cognitive self‐consciousness, FU = follow‐up, HADS = Hospital Anxiety and Depression Scale, MCQ‐30 = Metacognitions Questionnaire Short Form, MCQ‐A = Metacognitions Questionnaire (Adolescent Version), NC = need to control thoughts, NEG = negative beliefs about worry, POS = positive beliefs about worry, RCADS = Revised Anxiety and Depression Scale (Child Version).

^a^ Range: Children = 0–141; parents = 0–42.

^b^ Range: Children = 0–111; parents = 0–21.

^c^ Range: Children = 0–30; parents = 0–21.

* Significant group differences between mothers and fathers (*p* < 0.05).

### 3.2 Baseline Correlations

Bivariate correlations were used to explore and estimate relationships between child and parental symptoms, metacognitive beliefs and attention control beliefs at baseline. See Table 3. Correlations between children's and mothers' symptoms (*r*s = 0.10–0.22) as well as children's and fathers' symptoms (*r*s = −0.12 to −0.01) were non‐significant, with the exceptions being children's depressive symptoms and mothers' anxiety symptoms (*r* = 0.22, *p* < 0.05). Similarly, children's metacognitive beliefs were not significantly associated with mothers' (*r*s = −0.15–0.14) nor fathers' (*r*s = −0.23–0.20) metacognitive beliefs. Turning to attention control beliefs, only mothers' attention shifting significantly correlated with children's attention shifting and total attention control of a small magnitude (*r*s = 0.23, *p*s < 0.05).

**TABLE 3:** Bivariate correlations between child and parent measures at baseline.

		Mothers (*n* = 84)/fathers (*n* = 61)											
		HADS total	HADS anx	HADS dep	MCQ‐30 total	MCQ‐30 POS	MCQ‐30 NEG	MCQ‐30 CC	MCQ‐30 NC	MCQ‐30 CSC	ACS total	ACS focus	ACS shift
Child (*n* = 97)	RCADS total	0.152/−0.068	0.156/−0.109	0.106/−0.009	0.182/0.041	0.047/0.156	0.091/−0.046	0.179/−0.041	0.161/−0.017	0.211/0.080	−0.200/0.218	−0.134/0.211	**−0.222** */0.169
	RCADS anx	0.129/−0.061	0.126/−0.099	0.100/−0.008	0.160/0.030	0.025/0.121	0.073/−0.036	0.181/−0.035	0.136/−0.022	0.187/0.066	−0.190/0.189	−0.127/0.170	−0.212/0.157
	RCADS dep	0.189/−0.078	0.**217** */−0.123	0.101/−0.013	0.210/0.072	0.106/0.244	0.128/−0.071	0.132/−0.051	0.203/0.003	0.**239** */0.114	−0.186/0**.273** *	−0.129/0**.310** *	−0.204/0.182
	MCQ‐A total	−0.053/−0.057	−0.014/−0.116	−0.092/0.019	0.033/0.013	0.016/0.052	−0.097/−0.056	0.044/0.010	0.109/0.056	0.076/0.002	−0.077/0.144	−0.019/0.207	−0.111/0.067
	MCQ‐A POS	−0.111/−0.113	−0.056/−0.176	−0.157/0.022	−0.062/−0.167	0.038/0.048	−0.084/−0.179	−0.077/−0.066	−0.016/−0.094	−0.073/−0.234	−0.007/0.192	−0.030/0.114	0.012/0.198
	MCQ‐A NEG	0.080/−0.068	0.014/−0.163	0.149/0.049	0.056/0.084	−0.056/−0.016	−0.067/0.017	0.112/0.135	0.099/0.112	0.127/0.050	−0.034/0.069	0.051/0.154	−0.098/0.004
	MCQ‐A CC	0.041/−0.035	0.088/−0.013	−0.032/0.052	0.103/−0.084	0.139/−0.037	0.018/−0.119	0.073/−0.077	0.092/−0.133	0.091/0.060	0.041/0.143	0.049/0.237	0.029/0.045
	MCQ‐A NC	−0.125/0.038	−0.079/−0.044	−0.155/0.121	0.005/0.035	−0.016/0.093	−0.148/−0.050	0.011/0.007	0.138/0.117	0.068/−0.010	−0.096/0.030	0.003/0.113	−0.160/−0.033
	MCQ‐A CSC	−0.075/−0.038	−0.022/−0.043	−0.128/−0.024	0.011/0.168	−0.049/0.106	−0.077/0.114	0.038/0.037	0.080/0.203	0.059/0.125	−0.175/0.110	−0.133/0.152	−0.182/0.055
	ACS‐C total	0.029/0.052	−0.010/0.067	0.074/0.026	−0.106/−0.063	−0.106/−0.128	−0.060/0.093	−0.113/0.009	−0.042/−0.1069	−0.086/−0.130	0.170/−0.125	0.063/−0.017	0.**231** */−0.168
	ACS‐C focus	−0.030/0.091	−0.100/0.093	0.073/0.071	−0.117/−0.006	−0.128/−0.012	−0.095/0.109	−0.034/−0.014	−0.075/−0.016	−0.122/−0.091	0.139/−0.138	0.055/−0.047	0.185/−0.166
	ACS‐C shift	0.076/0.009	0.072/0.032	0.062/−0.018	−0.078/−0.101	−0.067/−0.206	−0.018/0.062	−0.163/0.028	−0.006/−0.102	−0.038/−0.140	0.168/−0.092	0.058/0.013	0.**230** */−0.140

Abbreviations: ACS = Attention Control Scale (Adult Version), ACS‐C = Attention Control Scale (Child Version), CC = cognitive confidence, CSC = cognitive self‐consciousness, HADS = Hospital Anxiety and Depression Scale, MCQ‐30 = Metacognitions Questionnaire Short Form (Adult Version), MCQ‐A = Metacognitions Questionnaire (Adolescent Version), NC = need to control thoughts, NEG = negative beliefs about worry, POS = positive beliefs about worry, RCADS = Revised Anxiety and Depression Scale (Child Version).

* *p* < 0.05.

Children's depressive symptoms were significantly and positively associated with mothers' cognitive self‐consciousness (*r* = 0.24, *p* < 0.05) while also being significantly associated with fathers' attention focusing and total attention control (*r*s = 0.27–0.31, *p*s < 0.05), that is, higher levels of children's depressive symptoms correlated with better perceived attention control in fathers. Finally, children's total level of symptoms was significantly and negatively associated with mothers' perceived attention shifting (*r* = −0.22, *p* < 0.05). The moderating effect of age on baseline child–parent correlations was tested for the bivariate associations that reached statistical significance. No moderating effect was found.

### 3.3 Do Parent Measures Predict Post‐Treatment Scores?

For the primary outcome (i.e., symptoms of anxiety and depression), results revealed no predictive effect of any parent measure (i.e., symptoms, metacognitive beliefs and attention control beliefs) at post‐treatment nor at 6‐month follow‐up. See Table 4. This was true both when investigating mothers and fathers. Effect sizes ranged from negligible to small at both time points (post‐treatment, *d*s = 0.04–0.21; 6‐month follow‐up, *d*s = 0.03–0.25). Turning to the secondary outcome (i.e., remission from primary diagnosis), results revealed few predictive effects of any parent measures, with the exception of fathers' attention control beliefs, which significantly predicted whether children remitted from their primary diagnosis at post‐treatment (*r* = 0.27, *p* = 0.034). Controlling for child baseline symptomatology, this result was only significant at the trend level (*p* = 0.053).

**TABLE 4:** Predictive effect of parent measures on post‐treatment measures.

		Primary outcome^a^						Secondary outcome^b^		
		Post‐treatment			6‐month FU			Post‐treatment		
		*t*	*p*	*d*	*t*	*p*	*d*	*B*	*SE*	*p*
HADS total	Mothers	0.475	0.636	0.10	0.650	0.517	0.14	0.031	0.049	0.528
	Fathers	−0.161	0.873	0.04	0.694	0.490	0.17	−0.105	0.078	0.179
MCQ‐30 total	Mothers	−0.917	0.362	0.20	−0.165	0.869	0.03	−0.004	0.019	0.834
	Fathers	0.252	0.802	0.21	1.019	0.312	0.25	−0.024	0.033	0.464
ACS total	Mothers	0.542	0.589	0.12	−0.285	0.776	0.06	0.014	0.026	0.595
	Fathers	−0.483	0.631	0.12	−0.856	0.396	0.21	0.112	0.053	0.**034** *

^a^ RCADS total score.

^b^ Remission from primary diagnosis based on ADIS‐IV‐C/P.

* *p* < 0.05.

## 4 Discussion

The present study investigated baseline associations between child and parental symptoms of anxiety and depression, metacognitive beliefs and attention control beliefs in a clinical sample of young people aged 10–17 years, and whether parental symptoms and higher‐order beliefs predicted MCT post‐treatment scores.

### 4.1 Intergenerational Associations Between Child and Parental Symptoms

No associations were found between child and parental symptoms of anxiety and depression at baseline, with the exception being children's depressive symptoms and mothers' anxiety symptoms. Yet, it should be noted that although the child sample in the present study did present with depressive symptoms (*M* = 11.75) above the 80th percentile when compared with normative data (Esbjørn et al. 2012), only a minority presented with a depressive disorder (10.3%, *n* = 10). The overall lack of statistically significant associations between child and parental symptoms is surprising given previous research suggesting that having a parent with an anxiety disorder is a robust predictor of the development of anxiety and depressive disorders in children (Creswell et al. 2020; Lawrence et al. 2019). Our finding could be explained in several ways. First, the parent sample was characterized by non‐clinical levels of anxiety and depression with mean scores falling within the normal range, suggesting that the majority of participating parents were experiencing good mental health. In turn, it is likely that our findings are influenced by a selection bias as treatment‐seeking samples possibly only represent a minority of clinical samples (Creswell et al. 2020; Rapee et al. 2023). For instance, lower levels of anxiety symptoms in mothers have previously been associated with increased odds of referrals for child treatment (Jongerden et al. 2015), and previous studies indicate systematic differences between young people seen at university or private clinics versus community clinics (Ehrenreich‐May et al. 2011; Southam‐Gerow et al. 2008). However, it is worth noting that although the parents reported non‐clinical levels of anxiety and depression, approximately 30% of mothers and 20% of fathers did report having experienced psychological problems within the past year. Therefore, we cannot rule out the possibility that parents under‐reported their symptoms due to social desirability (Paulhus 1991), for example, to look like good parents or due to fear of transgenerational transmission (Rapee 2000).

Nevertheless, it is important to consider that child and parental symptoms are not always systematically associated. Although we cannot rule out that a different pattern of results would have emerged in non‐treatment‐seeking samples or had the parent sample presented with higher levels of anxiety and depressive symptoms, our findings do not support the transgenerational transmissions of symptoms. Notably, only 49% of children with anxiety disorders have a parent with an anxiety disorder, and 65% of parents with an anxiety disorder have children *without* anxiety disorders (Rapee et al. 2023). Moreover, recent studies suggest that the strength of the association between child and parental symptoms differs depending on whether children's symptoms are assessed using self‐report or parent report, with the strongest effects found for the latter (e.g., Johnco et al. 2021; Köcher et al. 2023; Xerxa et al. 2021). For instance, in a recent study on children aged 8–16 years and their parents (Köcher et al. 2023), only parent‐reported child anxiety, but not self‐reported child anxiety, significantly correlated with parental anxiety. This was true both in a clinical (self‐report: *r* = 0.18, *p* = ns; parent report: *r* = 0.24, *p* < 0.05) and in a non‐clinical sample of children (self‐report: *r* = 0.24, *p* = ns; parent report: *r* = 0.52, *p* < 0.001). Thus, when children's symptoms are measured using parent report, it is possible that the observed child–parent associations are inflated by shared‐rater variance (Collishaw et al. 2009; Ringoot et al. 2015), that is, with some of the variance being a result of specific characteristics of the parent (e.g., the parent's world view or mental health) rather than a result of actual variance in the child's symptomatology.

### 4.2 Intergenerational Associations Between Child and Parental Higher‐Order Beliefs

Baseline associations between child and parental metacognitive beliefs assessed with the MCQ were non‐significant for both mothers and fathers. Although these findings are in line with the non‐significant associations found in a few previous studies of community samples (Donovan et al. 2017; Wilson et al. 2011), they contrast with the pattern of significant associations of a small to medium magnitude found in other community samples (Chow and Lo 2017; Esbjørn et al. 2016; Köcher et al. 2023; Lønfeldt et al. 2017), as well as preliminary findings in children with anxiety disorders (Köcher et al. 2023). Specifically, Köcher et al. (2023) investigated child–parent associations in the positive and negative metacognitive belief domains, finding significant associations of small effects (POS: *r* = 0.18, NEG: *r* = 0.24). In contrast, our results revealed negligible effects for these particular belief domains (POS: *r*s = 0.01–0.03, NEG: *r*s = 0.04–0.05). One explanation for this difference may be found in the use of different metacognitive measures. Whereas the Metacognitions Questionnaire for Adolescents (MCQ‐A; Cartwright‐Hatton et al. 2004) used in the present study consists of the original six items per subscale with four response options, the German adaptation of the MCQ for children (MKF‐K; Naumann 2014) used by Köcher et al. (2023) consists of five items per subscale and three response options only. Such differences in the measurement scales may influence scale properties and/or children's responses and make direct comparisons difficult. Another potential explanation of differences may be found in the relatively low mean levels of metacognitive beliefs (POS: *M* = 2.89, NEG: *M* = 5.87, range: 0–15) held by the clinical sample in the study by Köcher et al. (2023) compared to the sample in the present study (POS: *M* = 9.37, NEG: *M* = 16.90, range: 6–24) and other treatment‐seeking samples (e.g., POS: *M* = 7.98, NEG: *M* = 15.20, range: 6–24; Esbjørn et al. 2018). Additionally, Köcher et al. (2023) investigated child–parent associations for metacognitive beliefs in both a clinical and a non‐clinical sample with the largest effects found in the latter (POS: *r* = 0.54, NEG: *r* = 0.38). Thus, it is possible that the strength of the association between child and parental metacognitive beliefs depends on the level of child psychopathology and that parents play the most prominent role in children's early belief formation. Thus, future studies will need to evaluate associations between children's and parents' metacognitive beliefs and clarify whether the strength of this potential relationship differs depending on the level of child symptomatology.

With respect to attention control beliefs, overall associations between child and parent measures similarly revealed small and non‐significant associations, with the exception of mothers' attention shifting beliefs, which showed significant and positive associations with both children's overall attention control and attention shifting with small effects (*r*s = 0.23). This suggests a small positive relationship between child and maternal attention control beliefs, particularly with respect to the ability to flexibly shift attention, which is considered a key vulnerability factor in the S‐REF model. However, to the authors' knowledge, no study has previously investigated child–parent associations on measures of attention control beliefs, and therefore, the reliability of such effects needs to be investigated.

In sum, our results indicate only small or very weak child–parent associations, suggesting that other factors may be involved in the maintenance of metacognitive and attention control beliefs in clinical samples of young people. For instance, it is possible that parental behaviours previously linked to child anxiety and depression, such as over‐involvement (Yap and Jorm 2015) and parental control (Chyung et al. 2022; Van Der Bruggen et al. 2008), are associated with children's higher‐order beliefs and self‐regulation strategies as emphasized in the S‐REF model. For instance, there is preliminary evidence suggesting that parental criticism, rejection and discouragement are associated with biased metacognitive beliefs about rumination (e.g., ‘rumination helps me understand my past mistakes and failures’) in young people (age: 11–16, *r*s = 0.26–0.28; Chow and Lo 2017). However, it is also possible that we need to look beyond the role of parental factors, for instance, by considering the potential role of peers and social media (Rapee et al. 2019, 2023). Hence, studies investigating the influence of other parental and environmental factors on children and adolescents' higher‐order beliefs are warranted.

### 4.3 Predictive Value of Parental Factors on MCT Post‐Treatment Scores

Second, we investigated whether parental psychopathology, metacognitive beliefs and attention control beliefs at baseline predicted post‐treatment symptoms in children and adolescents following group MCT. Results showed that no of the included parent measures significantly predicted the primary treatment end point (i.e., symptoms of anxiety and depression) at post‐treatment nor at 6‐month follow‐up. The only predictive effect was found for fathers' total attention control beliefs, which significantly predicted the secondary outcome (i.e., remission from primary disorder) at post‐treatment. However, when controlling for children's baseline symptomatology, this result was only significant at the trend level and should therefore be interpreted with caution, given the overall pattern of null findings. The general lack of predictive effects could be explained in several ways. As previously mentioned, one explanation concerns a potential selection bias regarding the parent sample or parents having responded in socially desirable ways (e.g., to look like good parents). Another explanation could be that there simply are no or very weak predictive effects of parents' symptoms, metacognitive beliefs and attention control beliefs on MCT post‐treatment scores in children and adolescents. Nevertheless, this contrasts with previous research suggesting that parental psychopathology is a robust predictor of worse CBT treatment outcomes in children with anxiety disorders (Bertie et al. 2024a, 2024b; Hudson et al. 2015; Kunas et al. 2021). If replicated, this lack of effect has implications for treatment, suggesting that MCT outcomes in children and adolescents might be less affected by the mental health of their parents. However, findings from the present study should be considered preliminary, and future studies will need to further address the influence of parental factors as well as other variables potentially affecting MCT treatment gains. Indeed, given the previous research within CBT suggesting that other factors such as having multiple anxiety disorders, social anxiety and being older are predictors of treatment gains (Bertie et al. 2024a, 2024b; Hudson et al. 2015), future studies are encouraged to also consider the predictive value of non‐parental factors on MCT outcomes.

### 4.4 Limitations

The present study is not without limitations. First, it was based on secondary analyses of data from an uncontrolled trial. Given the small correlations observed between child and parental metacognitions in previous studies, our findings may be limited by the relatively small sample. In addition, we did not employ *p*‐correction as the study was not sufficiently powered to do so. Therefore, all significant results must be interpreted according to the effect size and must be repeated to ensure the robustness of our findings. Second, the parents were characterized by normal levels of psychopathology with a majority holding a medium to long education, suggesting a potential selection bias. Thus, future studies will need to address this potential issue, for instance, by recruiting families through community clinics rather than university and private clinics only. Third, the child sample consisted predominantly of females (82.5%) and a low percentage presented with a primary or comorbid depressive disorder (10.3%). Although anxiety problems are more common in children and adolescents than depressive problems, the generalizability of the results to populations of clinically depressed young people may be called into question. Fourth, although our results suggest limited influence of parental psychopathology and higher‐order beliefs, we cannot rule out the potential predictive value of other parental factors (e.g., parental behaviour) not included in the present study.

## 5 Conclusions

The present study showed predominantly small and non‐significant baseline associations between child and parental symptoms, metacognitive beliefs and attention control beliefs. There were, however, some exceptions; significant associations were found between maternal attention shifting and child attention shifting, total attention control and symptoms. Also, child depressive symptoms were significantly associated with maternal anxiety, maternal cognitive self‐consciousness and paternal attention control beliefs. Most of these relationships were small, apart from moderate associations between child depressive symptoms and paternal attention control. However, these findings need to be replicated and should be interpreted in light of the overall lack of other associations. Finally, our findings showed that baseline anxiety/depression symptoms, metacognitive beliefs, and attention control beliefs of parents did not predict MCT post‐treatment symptom scores in the children tested. The findings should be considered preliminary and interpreted in light of the small sample and the overall good mental health of the parents involved.

## Author Contributions

**Anne Thingbak:** conceptualization, methodology, investigation, formal analysis, writing – review and editing. **Jennifer L. Hudson:** conceptualization, methodology, writing – review and editing. **Adrian Wells:** conceptualization, methodology, writing – review and editing. **Mia Skytte O'Toole:** conceptualization; methodology, formal analysis, writing – review and editing.

## Ethics Statement

Ethical approval was obtained from the Regional Research Ethics Committee, and the study was conducted in accordance with the Helsinki Declaration II by the World Medical Association (WMA). The data were handled and protected in accordance with rules and regulations by the General Data Protection Regulation (GDPR) and the Data Protection Act.

## Consent

Custodians and children ≥ 15 years of age gave written informed consent, and children < 15 years of age orally assented to participate.

## Conflicts of Interest

Anne Thingbak, Jennifer L. Hudson and Mia Skytte O'Toole declare no conflicts of interest. Adrian Wells is the originator of MCT and a co‐director of the Metacognitive Therapy Institute.

## Data Availability

Data are available upon request to the corresponding author.

## Endnotes

^1^
While we initially planned to investigate parent variables both seperately for mothers/fathers and as a combined average for each parent unit, we decided to investigate only mothers and fathers seperately to reduce the number of analyses and preserve statistical power, given that the study was based on secondary analyses.
